# Probiotics as a Novel Treatment for Non-Alcoholic Fatty Liver Disease; A Systematic Review on the Current Evidences

**DOI:** 10.5812/hepatmon.7233

**Published:** 2013-04-09

**Authors:** Roya Kelishadi, Sanam Farajian, Maryam Mirlohi

**Affiliations:** 1 Faculty of Medicine and Child Growth and Development Research Center, Isfahan University of Medical Sciences, Isfahan, IR Iran; 2 Faculty of Nutrition and Food Sciences, Food Security Research Center, Isfahan University of Medical Sciences, Isfahan, IR Iran

**Keywords:** Non-Alcoholic Fatty Liver Disease, Probiotics, Fatty Liver

## Abstract

**Context:**

Non-alcoholic fatty liver disease (NAFLD) is a chronic liver disease, with 5-10% of liver having extra fat. Increase in its prevalence in all age groups is linked with obesity and Type II diabetes. The treatment of NAFLD remains controversial. A growing body of evidence suggests a relation between overgrowth of gut microbiota with NAFLD and non-alcoholic steatohepatitis (NASH). The objective of this review is to provide an overview on experimental and clinical studies assessing all positive and negative effects of probiotics.

**Evidence Acquisition:**

We made a critical appraisal on various types of documents published from 1999 to March 2012 in journals, electronic books, seminars, and symposium contexts including Medline, PubMed, and Cochrane Central Register of Controlled Trials databases. We used the key words: “non-alcoholic fatty liver disease, probiotics, non-alcoholic steatohepatitis, liver disease, and fatty liver”.

**Results:**

Probiotics, as biological factors, control the gut microbiota and result in its progression. It is in this sense that they are suggestive of a new and a natural way of promoting liver function. Correspondingly, limited evidence suggests that probiotics could be considered as a new way of treatment for NAFLD.

**Conclusions:**

Various experimental studies and clinical trials revealed promising effects of probiotics in improving NAFLD; however given the limited experience in this field, generalization of probiotics as treatment of NAFLD needs substantiation through more trials with a larger sample sizes and with longer-term follow up.

## 1. Context

Non-alcoholic fatty liver disease (NAFLD) is defined as a chronic condition with more than 5 to 10% of liver augmented by extra fat. The prevalence of NAFLD is rapidly rising, and is becoming a worldwide public health problem (1). Studies have confirmed that NAFLD is associated with body mass index (BMI), components of the metabolic syndrome, and insulin resistance (2). This disorder represents a spectrum of conditions ranging from fat accumulation alone (steatosis without inflammation) to steatohepatitis (NASH) with macro vesicular steatosis in hepatocytes, associated with inflammation and fibrosis (3). NAFLD occurs among all ages, both the genders and various ethnic groups, and its prevalence is reported to be 14-30% of the general population (4, 5). The prevalence of NAFLD is increasing as is the worldwide trend in obesity and Type II diabetes (6). Among the general adult population, the prevalence is estimated to be 20–30% and 15% in Western and Asian countries (4, 7, 8). Based on the Dallas Heart Study report, 30% of US adults are affected by NAFLD (9). A study on the US pediatric population reported a prevalence of 12.5% among children (10). In an Austrian study, 30-50% of obese children with different age and sex groups developed NAFLD; with age and sex considered as the two influential parameters on the prevalence of this disorder (11). In Italy, the corresponding figure was estimated to be 12.5% and 23% among general population and obese adolescents respectively (12). Limited study has been performed in the Eastern Mediterranean region and large epidemiological studies on the prevalence of NAFLD in the general population are scarce in Iran, with a prevalence rate of 2.8%-24% being reported in various age groups (2, 13, 14). Early studies indicated that NAFLD was more common in females (15) but recent studies have shown that NAFLD is almost evenly distributed among both genders, with a higher tendency among males (16). NAFLD is considered as one of the main causes of liver chronic disease along with obesity, diabetes and other components of the metabolic syndrome (17-19). The pathogenesis of NAFLD, and its development and progression to NASH remains to be determined. According to Day et al. (20, 21), the double ‘‘hit’’ theory can greatly explain the pathway and the mechanisms involved. At the ‘‘first hit’’, the development of simple liver steatosis occurs and is accompanied with systemic factors. Obesity and insulin resistance has been known to have a pivotal role in this phase (22). Such damage would result in increase in vulnerability to fatty liver changes (23) to subsequent inflammatory products like bacterial lipopolysaccharide (LPS) (22). The main factors contributing to initiation of the second hit are oxidative stress, subsequent lipid peroxidation, produced pro-inflammatory cytokines, such as tumor necrosis factor (TNF) α (24, 25), and hormones secreted from adipose tissue (26, 27). All these factors aggravate the transformation of NAFLD to NASH or even to cirrhosis. Currently, there is limited proven effective treatment for NASH. Since its underlying cause and its prognosis are not well understood, therapeutic modalities considered for patients with NAFLD has typically been focused on the management of associated conditions such as obesity, diabetes mellitus, and hyperlipidemia (28). The main treatment for NAFLD is lifestyle modification, including weight loss through a combination of decreased energy intake and increased energy expenditure (29).

Recent studies have reported a possible impact of gut micro flora overgrowth on the development of NAFLD/NASH (30, 31). One trillion microorganisms inhabit the human gut, and gut micro flora have a dual impact on the liver function. The fermentation products such as ethanol, ammoniac and acetaldehyde produced by gut microflora are metabolized in the liver. Moreover, lipopolysaccharide from gram negative gut micro flora is continuously released after bacterial death as endotoxin and transported via a toll-like receptor 4 (TLR-4)-dependent process into intestinal capillaries (19). This induces cytokines formation and secretion from liver (28). It is suggested that liver injury and fibrosis could be partly caused by exposure to bacterial products like LPS (32). TLR4-bearing stellate cells respond to LPS, producing inflammatory cytokines and chemokines, but also promoting fibrosis. Further research supports the role of TLR4 in promoting fibrosis. It has been shown that deficiency in myeloid differentiation factor-2 (MD-2), the coreceptor of TLR4, and TLR4 expression, may attenuate liver inflammation and fibrosis in mice affected by NASH (32). A study reported significantly higher counts of Escherichia-coli and staphylococcus in the stool samples of patients with mild encephalopathy and cirrhosis than in healthy controls (33). The relation between gut micro flora and NAFLD might be mostly because of the endogenous LPS (34). Gut micro flora have important role in the host physiological function and metabolism through the following mechanism: conversion of pro-carcinogen into less harmful substances, production of vitamins, degradation of bile acid and cholesterol, as well as facilitating nutrient digestion, especially fermentation of non-digestible carbohydrates (35). According to the currently adopted definition by the Food and Agriculture Organization and World Health Organization (FAO/WHO), probiotics are: Live microorganisms, when administered in adequate amounts confer a health benefit on the host (36). Probiotics are used as effective biological factors for modulation of gut micro flora, and recently they are suggested as natural means for improving the liver function (35). The colonized gut flora in healthy individuals are affected by many physiological and environmental factors e.g., nutrition, illness, aging, and stress are mainly discussed in the literature as the key parameters affecting the imbalance and impairment of the natural pattern of gut mechanism in healthy individuals. Therefore, probiotics are suggested as a beneficial agent to better balance the gut flora. Because of the functional link to the intestine, the liver is known as the first organ barrier against the gut-derived bacterial fractions or metabolites, which are persistently released in to the circulation. The kupffer cells as the liver macrophages particularly reduce the amount of bacterial phagocytosis and endotoxins (20). The application of probiotics as suggested treatment for NAFLD has led to new insight in this field. However, there is limited experience and experimentation to corroborate this concept. The present study aims to conduct a systematic review on both experimental and clinical trials performed on the effects of probiotics on NALFD to date.

## 2. Evidence Acquisition

By conducting a critical appraisal, we reviewed various types of study designs, i.e., prospective cohort study, clinical trials, cross-sectional studies. We focused on the available sources between 1999 and 2012, including journals, electronic books, seminars, and symposium contexts, Medline, PubMed, and Cochrane Central Register of Controlled Trials databases. We used the key words: "non-alcoholic fatty liver disease, probiotics, non-alcoholic steatohepatitis, liver disease, and fatty liver". The search was limited to English language, and the relevance of surveys was found using a hierarchical approach based on the titles, abstracts, and full text of published articles. The information was summarized based on the following key indicators: lead author, year of publication, sample size, experimental model, follow-up duration, and outcome measure. In the initial search, we found 244 entries on liver disease and probiotics, 70 articles on fatty liver and probiotics, 14 entries on NASH and probiotics, and 11 articles on NAFLD and probiotics. After compiling the complete list of articles, they were screened based on their target group, i.e., the animal and human studies. Scanning the articles was constructed on the basis of the title, abstract, and their major purposes which were limited to NAFLD biomarkers and consumption of probiotics.

### 2.1. Animal Studies

Tracing the NALFD response to probiotic intervention in animal models for histological lesions, fatty liver, inflammation, fibrosis, and the metabolic disturbances associated with NAFLD (32) were conducted. Then the process of improvement after probiotic intervention was explored. The results from the animal models were divided into two of the following categories:

1) Models were used where NALFD was induced by a modification to the animal's diet (37). Over-nutrition of a high fat diet (HFD), containing (40-70%) fat, results in obesity-induced NALFD, and subsequent inflammation as well as an increase in TNFα concentration. In some studies, the application of such diets is combined with the use of genetically hyperphagic animals.

The disadvantages to this method is that the results can be affected by the quantity and quality of the fat in the given test diet, the animals species being tested and the duration of the study.

The signs of NALFD also can be induced using methionine- and choline-deficient (MCD), or atherogenic diets containing 2-5% cholesterol and cholic acid.

2) Providing genetically modified mice (37) with NALFD is an alternative model to trace the response to probiotic intervention. Mutation at receptor (db/db and fa/fa) or mutation at the ligand (ob/ob) leads to disturbance in leptin signaling pathway that results in a similar phenotype as NALFD. Moreover, it is shown that overexpression of transcription factor SREBP-1c as well as heterozygous mutation on the agouti gene (KK-Ay/a) results in impaired IKK/NF_B signaling pathways culminated in similar, but not identical, phenotypes to NALFD. In a four-week study, Li et al. (38) showed that the administration of a diet containing a commercial probiotic type (VSL#3) to the ob/ob mice fed with HFD affected their obesity status and liver histology in a positive manner. Moreover, a decrease in the liver’s total fatty acid and serum alanine transaminase (ALT) was observed. These changes were attributed to the anti-tumor necrosis factor α (TNFα) properties of consumed probiotic (3, 39, 40). The reduction of B- oxidation of fatty acids as well as decrease in insulin resistance markers like nuclear factor kappa-light-chain-enhancer of activated B cells (NFκB), were spotted in this study report. Furthermore, the reduction of TNF-α which has an important role on insulin resistance in NAFLD patients, was seen as the reason for the observed changes (41, 42). In a four-week animal study, Ma et al. (43) used the same probiotic prepration (VSL#3), and HFD-induced steatosis and insulin resistance in the test species decreased significantly. It is suggested that HFD leads to the depletion of natural killer T cells (NKT) and caused an increase in TNFα and inflammation (3). The results in the latter study were attributed to the increase in NKT due to probiotic consumption. NKT are unconventional T cells that express both T cell and Killer cell receptors. They regulate hepatic inflammatory process through balance in the production of pro- and anti-inflammatory cytokines. Alterations of NKT function might lead to overproduction of TNF-α, causing inflammation and insulin resistance (44). Likewise, in another study carried out by Esposito et al. in Italy (3), the probiotic mix VSL#3 was shown to be beneficial in the alleviation of NALFD symptoms. A 4-week study on rats showed improvement in the liver inflammation; steatosis, peroxidation indexes, and a decrease in ALT concentration in HFD mice. Moreover, the study showed that administration of VSL# 3 in the mice diet resulted in the improvement of the lipid profile and a decrease in oxidative damage, protein nitrosylation, TNFα and NFκB concentrations. It is suggested that the given NFκB reduction led to the decline in inducible nitric oxide synthases (iNos), and Cyclooxygenase 2 (COX2) (2). Furthermore, the decreased level of Peroxisome proliferator-activated receptor (PPAR-α) concentration through HFD, which is regarded as a cause of liver steatosis, was observed as well. In addition, the increased level of macrophages and lymphocytes returned to normal status by probiotic intervention (45). In a nine week study, VSL#3 was used as a probiotic supplement for methionine- and choline-deficient (MCD) rats (46) with insulin resistance, although the MCD-induced steatosis and inflammation were not statistically influenced by probiotic intervention, but improvement in liver fibrosis was observed. This study was a long-term study as opposed to the other studies mentioned above. There is a good chance that the difference in the NALFD inducer diet (using MCD instead of HFD) may explain the difference in the findings. Severe atrophy of adipose tissue due to application of MCD showed that NASH had stronger association with lipodystrophy than with metabolic syndrome (47). As well, in another study conducted by Karahan et al. in Turkey, MCD induced steatohepatitis rats were treated by Pro 1 ( included Lactobacillus fermentum (BB16-75, AK2-8, AK5-22, AK6-26), Lactobacillusplantarum (AA17-73, AK7-28, AK8-31B) and Enterococcus faecium (AB6-21, AB16-68, AK4-120, AK7-31, BK9-40, BK13-54)) and Pro 2 (consisted of six bacterial strains (Enterococcus faecium BK10-47 and Lactobacillus plantarum AB7-35, AC3-16, AC21-101, AB16-65, BK10-48)) during two- and six-week intervals. It showed a decrease in the incidence of steatohepatitis in at least 50% of the rats in both the short- and long-term studies. The preventive effect of probiotics may be due, in part, to modulation of apoptosis and their anti-inflammatory activity (48). In the study of Lee at al., an 8 week intervention of giving L.rhamnosus to HFD rats led to improvement of liver steatosis, with a strong effect on weight loss (49). Another organism used in this field is Lactobacillus plantarum (50). Its use during five weeks in rats fed with high cholesterol diet showed a decrease in the liver cholesterol and triglycerides content. In this study the amount of lactobacillus and bifidius increased in stool samples. Another experimental study conducted by this probiotic using a period of 14 weeks resulted in decreasing levels of cholesterol, LDL-C and triglycerides, but without any effect on HDL-C (51). During eight week treatment with L.casei and L.acidophillus, improvement was shown in insulin resistance and a reduced level of liver stress oxidative. Rats’ diets in this study induced diabete mellitus with insulin resistance, hyperinsulenimia and increased storage of fructose. Probiotic therapy in these rats resulted in improvement of insulin resistance and also a reduction in the liver insulin and glycogen levels. It would seem that malondialdehyde (MDA) level as marker of oxidative stress also showed that these two probiotics could improve lipid peroxidation level (52).

The study of Paik *et al*., six weeks of giving Bacillus polyfermenticus to HFD and high cholesterol diet to rats showed improvement on the level of liver and serum lipid profile, especially on total- and LDL-cholesterol levels (53). A summary of the effects of probiotics on NAFLD in experimental studies is presented in Table 1.

**Table 1.** Summary of the Effects of Probiotics on NAFLD in Experimental Studies

Results	Experimental Model	Duration of Therapy	Reference
**Reduction in hepatic total fatty acid content, and serum ALT^a^ levels**	Mice: ob/ob mice fed HFD	4 weeks	Li *et al*. (2003) (38)
**Improved hepatic steatosis and inflammation, reduction in liver and serum cholesterol and triglycerides**	Mice: HFD	4 weeks	Ma *et al*. (2008) (43)
**Improved in hepatic inflammatory, steatotic and peroxidative factors and reduction in serum aminotransferase levels**	Rats: HFD	4 weeks	Esposito *et al*. (2009) (3)
**Amelioration of liver fibrosis and steatosis**	Mice: MCD	9 weeks	Velayudham *et al*. (2009) (46)
**Improved hepatic steatosis**	Mice: HFD	8 weeks	Lee *et al*. (2006) (49)
**L. plantarum MA2 feeding lowered serum total cholesterol, LDL-C, and triglycerides level, without change in HDL-C**	Rats: cholesterol-enriched diet	5 weeks	Wang *et al*. (2009) (50)
**Reduction in cholesterol, LDL-C and triglycerides , no effect on HDL-C**	Rat	14 weeks	Fazeli H *et al*. (2010) (51)
**Reduced liver oxidative stress and insulin resistance**	Rats: high-fructose diet	8 weeks	Yadav *et al*. (2007) (52)
**Reduction in plasma LDL-C, cholesterol, and hepatic total cholesterol, and triglycerides**	Rats: high-fat and high-cholesterol diet	6 weeks	Paik *et al*. (2005) (53)
**An insignificant decrease in serum ALT levels of rats fed MCD MCD elicited TNF-α, Bcl-2, caspase 3 and caspase eight expressions in all rats. reduce steatohepatitis**	Rats: MCD	2 weeks + 6 weeks	Karahan *et al.* (2011) (48)

^a^Abbreviations: ALT, alanine transaminase; HDL-C, high-density lipoprotein-cholesterol; HFD, high fat diet; LDL-C, high-density lipoprotein-cholesterol; MCD, methionine and choline-deficient (MCD); NAFLD, non-alcoholic fatty liver disease.

### 2.2. Human Studies

In our review of the literature, we found few human trials studying the effects of probiotics on NAFLD (Table 2). In the study of Loguercio et al. in 2005, VSL#3 was administered for three months in four groups of patients. This trial comprised patients with NAFLD (n = 22), alcoholic fatty liver (n = 20), chronic hepatitis C (n = 20), and cirrhosis (n = 16). The results showed improvement in markers of lipid peroxidation, i.e., MDA; 4-hydroxynonenal (4-HNE) in NAFLD and alcoholic fatty liver patients. In addition, a reduction of TNF-α،IL6،IL10 was observed in alcoholic fatty liver patients. In all of the patients recruited to this trial, S-NO levels were reduced after the intervention (54). The same scientific team conducted a pilot study on NAFLD patients, and studied the effects of probiotics on chronic liver disease (55). They studied three following groups of patients: patients with chronic hepatitis C (n = 12), alcoholic fatty liver (n = 10) and NASH (n = 3). They were given Lactobacillus acidophilus, bifidus, rhamnosus, plantarum, salivarius, bulgaricus, lactis, casei, breve mixed with prebiotic Fructooligosaccharide (fos) and vitamins as B2, B12, B6, D3, C, and folate. After two months, serum concentrations of MDA،4HNE،TNF-α, and ALT decreased in NASH patients. A recent Spanish study evaluated the effects of an acute treatment with a mixture containing 500 million of Lactobacillus bulgaricus and Streptococcus thermophilus per day in patients with NAFLD. The study showed a reduction in ALT and aspartate aminotransferase (AST) activity and and gammaglutamine transferase (Gamma GT) levels; however, in groups, the anthropometric parameters and cardio-metabolic risk factors remained unchanged after treatment (56). A pilot study on NAFLD patients in Johns Hopkins hospital on four adults showed a significant increase in liver fat at the end of four months. However, the investigators themselves declared that their study had multiple limitations, including the fact that they only evaluated one dose and preparation of the probiotic compound (55).

**Table 2.** Summary of the Effects of Probiotics on NAFLD in Clinical Trials

Results	Country	Probiotic	Population	Reference
**In NAFLD and AC^a^ groups, VSL#3 improved plasma levels of lipid peroxidation markers: MDA, 4-HNE. In AC patients, cytokines (TNF-α, IL-6 and IL-10) improved. S-NO plasma levels improved in all groups**	Italy	VSL#3	NAFLD, AC, chronic hepatitis C, liver cirrhosis	Loguercio *et al.* (2005) (54)
**Decreased serum ALT, MDA, 4-HNE and TNF-α in NASH patients**	Italy	LAB associated to prebiotics (FOS) and vitamins (B6, B2, B12, D3, C and folic acid)	chronic hepatitis, C, AC, NASH	Loguercio *et al.* (2002) (55)
**Decreased serum ALT, AST, Gamma GT**	Spain	Lactobacillus bulgaricus and Streptococcus thermophilus	NAFLD	Aller *et al.* (2011) (56)
**A significant increase in liver fat observed; there were no significant differences in glycosylated hemoglobin TNF-α, IL-6, and interferon g), and height, weight, body mass index, and medication use)**	US	VSL#3	NAFLD	Solga *et al.* (2008) (72)

^a^Abbreviations: AC, alcoholic liver cirrhosis ALT, alanine transaminase; AST, aspartate aminotransferase; Gamma GT, gammaglutamine transferase; HNE, hydroxynonenal; MDA, malondialdehyde; NAFLD, non-alcoholic fatty liver disease; NASH, non-alcoholic steatohepatitis.

## 3. Results

The summary of the effects of probiotics on NAFLD in experimental studies is presented in Table 1, and the corresponding figure for clinical trials in Table 2.

## 4. Conclusion

Reviewing the human and animal studies revealed an affirmative effect of probiotics supplementation on markers of NAFLD and NASH (3, 38, 43, 45, 47, 54). The general effect of probiotic in this regard seem to be related to reducing the impact of pathogenic bacteria on NAFLD development by exclusion or inhibition of invading bacteria, as well as by producing antimicrobial factors such as SCFA (58). These mechanisms also resulted in an improvement of gut micro flora (59-64). SCFA produced by Lactobacillus have an important role in reducing pH of the gut and finally in inhibiting the growth of gram negative bacteria (61). Furthermore, probiotics can modify the epithelial permeability and function, and are capable of improving the non-specific intestinal barrier defense by stimulating the production of tight junctional protein mucins. This adversely affects the small intestinal bacterial overgrowth and bacterial translocation. Moreover, as the important product of probiotics, SCFA can finally modify the epithelial permeability (62-64). Studies conducted on Lactobacillus GG, Bifidobacterium infantis, Bifidobacterium lactis and Escherichia coli Nissle showed that these probiotics may increase the tight junction integrity (65, 66). On the other hand, establishment of probiotics in the gut decreases endotoxemia. After the death of gram negative bacteria, LPS is released into the intestine and penetrates into the gut capillary through TLR-4 and in turn, provokes TNFα (23). Consequently, this probiotic may suppress inflammation. Intestinal inflammation leads to an increase of mucosal permeability and bacterial translocation. Several cytokines, such as TNF-α have been shown to increase the permeability in the invitro condition; altering tight junction morphology and distribution thereby creating a self-perpetuating vicious cycle (66). Some studies proposed the anti-inflammatory effects of probiotics like VSL#3 and Lactobacillus reuteri are through suppressing IL-8 secretion, which is a potent neutrophil-activating chemokine, and is released from intestinal epithelial cells in response to several pathogenic bacteria (67). IL-8 is transcriptionally regulated by NF-κB. Given that obesity has become such a pervasive health problem at both individual and public health levels (68-70), combined with the environmental pollution with NFLD (1), a rapid increase in the prevalence of NAFLD is expected in the near future. Thus, finding safe and appropriate treatment modalities is warranted for NAFLD and probiotics may be considered a promising therapy in this regard. Several studies in this review support the probable role of probiotics in treatment of liver disorders especially NAFLD and NASH. There are a few trials with limited number of participants in this field, but studies showed a 100% reduction in MDA, 4-HNE levels as lipid peroxidation markers and inflammatory cytokines like TNF-α. The review of nine animal studies showed that steatosis improved in 44% of cases and liver enzymes levels and serum cholesterol and triglycerides concentrations were reduced in 22% cases. Additionally, in 11% of cases the liver fatty acids decreased, and in 22% of cases, improvement in liver tissues and fibrosis was observed. Besides the effect of probiotics on inflammation status in NAFLD patients, this supplementation may also influence lipid reduction. The mechanisms explaining this observation may be as follows: i. fermentation products of lactic acid bacteria inhibit cholesterol synthesis enzymes and thus reduce cholesterol production; ii. The bacteria may facilitate the elimination of cholesterol in feces; iii. By binding with cholesterol, the bacteria may inhibit its reabsorption into the body; IV. The interference of bacteria with the recycling of bile salt (a metabolic product of cholesterol) may facilitate its elimination; and v. the assimilation of lactic acid (71, 72). All of these findings are promising for the use of probiotics as treatment of NAFLD. For further clinical investigation, it is necessary that trials with large sample sizes and long-term follow-up are performed to assess all possible effects and side effects of probiotics. Furthermore, in this kind of treatment, it is important to pay attention to the strain of probiotics, the trial duration, the accompaniment of probiotics with prebiotics and the duration of the viability of bacteria in the gut after administration, because any change in these factors could have an effect on the use of supplementation. Expanding the probiotics as treatment for NAFLD is promising, but again, its clinical use warrants more trials with large sample sizes and longer-term follow-up.
